# Impact of the control for corrupted diffusion tensor imaging data in comparisons at the group level: an application in Huntington disease

**DOI:** 10.1186/1475-925X-13-128

**Published:** 2014-09-01

**Authors:** Hans-Peter Müller, Jan Kassubek, Georg Grön, Reiner Sprengelmeyer, Albert C Ludolph, Stefan Klöppel, Nicola Z Hobbs, Raymund AC Roos, Alexandra Duerr, Sarah J Tabrizi, Michael Orth, Sigurd D Süssmuth, G Bernhard Landwehrmeyer

**Affiliations:** Department of Neurology, University of Ulm, Ulm, Germany; Section Neuropsychology and Functional Imaging, Department of Psychiatry, University of Ulm, Ulm, Germany; School of Psychology and Neuroscience, University of St Andrews, Scotland, UK; Department of Psychiatry and Psychotherapy, Section for Gerontopsychiatry and Section of Experimental Neuropsychiatry, University Medical Center Freiburg, Freiburg, Germany; Department of Neurodegenerative Disease, UCL Institute of Neurology, London, UK; Department of Neurology, Leiden University Medical Centre, Leiden, The Netherlands; Department of Genetics and Cytogenetics, and INSERM UMR S679, APHP, ICM Institute, Hôpital de la Salpêtrière, Paris, France

**Keywords:** Corrupted raw data, Diffusion tensor imaging, Fractional anisotropy, Huntington disease, Multicenter study

## Abstract

**Background:**

Corrupted gradient directions (GD) in diffusion weighted images may seriously affect reliability of diffusion tensor imaging (DTI)-based comparisons at the group level. In the present study we employed a quality control (QC) algorithm to eliminate corrupted gradient directions from DTI data. We then assessed effects of this procedure on comparisons between Huntington disease (HD) subjects and controls at the group level.

**Methods:**

Sixty-one HD patients in early stages and forty matched healthy controls were studied in a longitudinal design (baseline and two follow-ups at three time points over 15 months), in a multicenter setting with similar acquisition protocols on four different MR scanners at four European study sites. A QC algorithm was used to identify corrupted GD in DTI data sets. Differences in fractional anisotropy (FA) maps at the group level with and without elimination of corrupted GD were analyzed.

**Results:**

The elimination of corrupted GD had an impact on individual FA maps as well as on cross-sectional group comparisons between HD subjects and controls. Following application of the QC algorithm, less small clusters of FA changes were observed, compared to the analysis without QC. However, the main pattern of regional reductions and increases in FA values with and without QC-based elimination of corrupted GD was unchanged.

**Conclusion:**

An impact on the result patterns of the comparison of FA maps between HD subjects and controls was observed depending on whether QC-based elimination of corrupted GD was performed. QC-based elimination of corrupted GD in DTI scans reduces the risk of type I and type II errors in cross-sectional group comparison of FA maps contributing to an increase in reliability and stability of group comparisons.

## Background

Diffusion tensor imaging (DTI) has become increasingly accepted in magnetic resonance (MR) neuroimaging [1, 2]. As with other MR modalities, the quality of diffusion weighted images (DWI) can be affected by a variety of factors, such as acquisition sequence, homogeneity of the magnetic field, stability of the gradient amplitude, slew rate variations, as well as by differences in multichannel radio-frequency coils and parallel imaging parameters [2]. The acquisition time for DTI is longer than for conventional MR imaging due to the large number of recorded gradient directions (GD) that are required. Artifacts in GD may originate from both the acquisition system (such as eddy-current and vibration artifacts) and the subject scanned, such as cardiac pulsation and particularly head motion [2, 3]. Signal changes produced by these artifacts can be severe and may eventually result in erroneous diffusion tensor values [4]. In order to detect artifacts in DTI, quality control (QC) assessment was suggested in previous studies with a variety of methods [2, 4–7]. In 2007, quality assessment was performed by Hasan [5] using isotropic tensor scans of water phantoms providing a useful framework for QC and parameter optimization in DTI. In vivo DTI QC was performed by the software tool DTIprep [4] which provides a framework for automatic QC by slicewise correlation check. Most recent studies introduced a sophisticated QC method for detecting bias of Fractional Anisotropy (FA) and the principal direction by a Rician noise model [2] or suggested an integrative tool for an automatic DTI analysis and quality assurance pipeline [7]. However, the effect of corrupted volumes within DTI data sets on the results of comparisons at the group level has not been investigated yet.

The aim of this study was therefore to examine if the application of QC to DTI data, in combination with strict elimination of corrupted GD, results in differences in group comparisons of DTI-based metrics compared to using no QC. To this end, we used data from patients with Huntington disease (HD) who are prone to involuntary movement during a DTI scan. We used data from the PADDINGTON study (Pharmacodynamic Approaches to Demonstration of Disease-modification in Huntington’s disease by SEN0014196), a multi-centre study with a total sample size of initially 101 data sets. In addition, participants underwent MRI three times 6 months apart, which allows validation of the results, i.e., results clusters for comparisons at the group level.

HD is a monogenetic, autosomal dominant neurodegenerative disorder of high penetrance characterized by a progressive dysfunction of the basal ganglia, clinically resulting in cognitive decline, typically hyperkinetic movement disorder and behavioural alterations (e.g. apathy, perseveration and irritability). Demonstrating biological alterations during the pre-clinical phase in HD mutation carriers may permit the identification of biomarkers, and any modulation of such potential biomarkers by interventions with therapeutic intent may point to the possible efficacy of experimental therapies. Therefore, neuroimaging techniques investigating structural and functional properties of the HD brain may have biomarker potential (for reviews see [8, 9]): given the pathophysiology of HD and its effects on white matter integrity an outstanding representative of these potential technical biomarkers is DTI.

In the present study, those structures were of particular interest that had been identified in previous DTI studies in HD: an increase of FA values in the basal ganglia, and FA reductions in the external and internal capsule, in parts of the thalamus, and in subcortical white matter [10–12]. The present study addresses the investigation of the impact of QC on DTI data with the exemplary application to HD. We investigated whether identification and elimination of corrupted GD from cross-sectional data sets would lead to changes in cross-sectional result patterns. Therefore, the novelty of this study is to examine if and to which extent disturbances during DTI acquisition had an effect on DTI-based metrics at the group level. The main question was whether between-group differences are reliable with or without application of QC, i.e. does the application of QC have any effect on the results of between-group differences. While previous studies had investigated the impact of QC on single subject data, we extended this concept to a study at the group level. Hence, this study is a continuation of previous QC-studies in DTI [2, 4–7] and complements those studies for aspects of QC on the reliability of between-group statistics.

## Methods

### Subject scanning

Data in this study were collected in the framework of the European PADDINGTON project at four study sites in Europe (Leiden, the Netherlands; London, UK; Paris, France; Ulm, Germany) [13]. The PADDINGTON study is an international initiative that aims to provide pharmacodynamic approaches for disease-modifying clinical trials. Work package 2 of this project entails the collection of 3.0 *Tesla* MRI (volumetric and DTI) scans acquired using similar acquisition protocols from patients with HD in an early disease stage and from healthy control participants, with the objective of identifying biomarkers of disease progression. The study was conducted in accordance with the Declaration of Helsinki and the International Conference on Harmonisation guideline on Good Clinical Practices and applicable local regulatory requirements and laws. All participants were ambulatory and agreed to volunteer for MRI scanning after giving written informed consent.

All HD patients had a genetically confirmed diagnosis with a trinucleotide (cytosine-adenine-guanine) repeat length of 36 or higher, and had clinical features of mild HD at stage I based on the Unified Huntington’s Disease Rating Scale (UHDRS) with a Total Functional Capacity (TFC) score of 11–13. In total, 61 HD and 40 control subjects were scanned at visit 1, 56 HD and 39 control subjects were scanned at visit 2 (6 months after baseline), and 55 HD and 37 control subjects were scanned at visit 3 (15 months after baseline).

Acquisition parameters for the different sites were similar with slight variations of the standardized acquisition protocol. DTI was performed with echo planar sequences, where each data volume consisted of 52 to 76 axial slices of 2.0 mm or 2.2 mm thickness (depending on the scanner of the different sites, whole brain coverage was guaranteed), with no inter-slice gaps, and an acquisition matrix of between 112 ×112 to 128 × 128 with in-plane resolution of 2.0 × 2.0 mm^2^, or 2.2 × 2.2 mm^2^, respectively. TR ranged between 8 s and 13 s, and TE ranged between 56 ms and 86 ms. Each DTI data set consisted of more than 40 b = 1000 s/mm^2^, and one or more b = 0 scans. More detailed acquisition parameters for the different sites have already been reported previously [12].

### Diffusion tensor imaging and data analysis overview

A DTI scan consists of a number of gradient encoding volumes, e.g. some b = 0 scans as well as a number of scans with different diffusion encoding gradients [14]. Diffusion tensor calculation results in an over-determined equation system and further parameterization for quantification of the diffusion anisotropy is the fractional anisotropy (FA) [15]. FA, a dimensionless scalar measuring the diffusion directionality in a single voxel, was used as the DTI-based metric for this study.

In a general simplification, any measured MR signal is a combination of the true quantity, acquisition system noise, environmental noise, and subject specific noise. Ideally, the quality of the measured signal (i.e. the signal to noise ratio: SNR) can be improved by signal accumulation [16]. If applied to DTI data, the repeated recording of diffusion encoding volumes should lead to an improvement of the accurateness of the diffusion tensor and the FA value in each voxel [17, 18]. Thus, if some of those repeated gradient direction (GD) images were corrupted by subject movement or by other sources of noise, the integration of these corrupted GD images into the tensor calculation (or FA values, respectively) would bias the results. Contrariwise, the removal of noisy or corrupted volumes from tensor calculation (or FA values, respectively) could reduce this bias [6].

**Figure 1:**
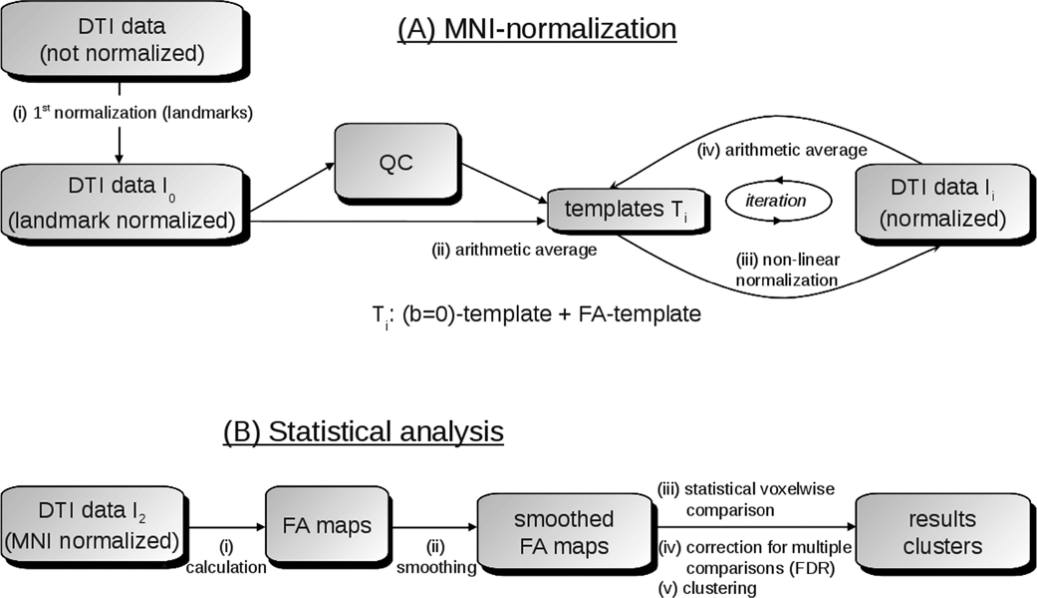
**Analysis schemes for cross-sectional comparison. (A)** Schematic example for an iterative template-specific MNI-normalization: after a 1^st^ normalization step based on landmarks, first templates T_1_ ((b = 0) template and FA-template) were obtained by arithmetic averaging of DTI-data I_0_. Analyses were performed with or without quality control (QC) and subsequent gradient direction elimination. Subsequently, in an iterative procedure, normalized DTI-data I_1_ were obtained by non-linear normalization to the previously defined templates (T_1_). From these newly normalized DTI-data I_1_, new templates (T_2_) were derived which again could be used for normalization. This iterative process is stopped when a predefined coincidence (measure by correlation) between DTI-data and templates was reached. **(B)** Scheme for whole brain-based spatial statistics: FA-maps are calculated from normalized DTI data and a smoothing filter to the individual normalized FA-maps is applied. In a consecutive step, voxelwise statistical comparison between the patient groups and the corresponding control group is performed. Final steps are correction for multiple comparisons using the false-discovery-rate (FDR) algorithm and a clustering procedure for further reduction of type I and type II errors.

The in-house DTI analysis software *Tensor Imaging and Fibre Tracking* (TIFT; [19, 20]) was used for post-processing and statistical analysis. Figure 1 shows a schematic overview of data processing and analysis, divided into the iterative template-specific normalization to the Montreal Neurological Institute (MNI) stereotactic frame [21] – with and without QC, respectively (Figure 1A), and the scheme for statistical analysis (Figure 1B). Whole brain-based spatial statistical analysis is a voxel-based DTI analysis approach in which unbiased results at whole brain basis are obtained, as previously reported [20, 22]. FA-maps were calculated from MNI-normalized DTI data, and a Gaussian smoothing filter of 8 mm FWHM was applied to the individual normalized FA-maps. In a consecutive step, voxelwise statistical comparison between the patient groups and the corresponding control group was performed by Student’s t-test. FA values below 0.2 were not considered for calculation as cortical grey matter shows FA values up to 0.2 [23]. Next steps were correction for multiple comparisons using the false-discovery-rate (FDR) algorithm [24] at p < 0.05, and a clustering procedure for further reduction of type I and type II errors. In general, a threshold cluster size of 512 voxels is to be considered a good choice [12]. However, a lower threshold cluster size of 64 voxels (corresponding to a sphere with radius of approximately 1 acquisition voxel) was applied in this study in order to elucidate also small-size differences.

### Quality Control (QC)

For each GD *j*, the weighted intensity difference of each slice *n* to the respective slice of all other GD was computed
1

where

〈*a*_*j*,*n*_〉 denotes the arithmetic average intensity of the slice *n* under observation of GD *j* and 〈*a*_*i*,*n*_〉 slice *n* for comparison of GD *i.*

The relative average intensity deviation for slice *n* was then weighted by the dot product of vectors of two GD *i* and *j*, , summarized for all GD *i* and subtracted from 1:
2

reflecting the deviation of a single slice *n* of GD *j* to the respective slices of all other GD.

A global quality quantity for GD *j* could be defined as the minimum of *diff*(*I*_*j*,*n*_) for all slices n:
3

*Q*_*j*_ reflects the minimum of slicewise comparisons of all slices for GD *j*. If *Q*_*j*_ falls below a certain threshold, the whole GD was eliminated for analysis. The procedure is not iterative, and *Q*_*j*_ values of different GD are influenced by each other. Thus, in the case of a series of corrupted GD (with hypointense slices), the global level of *Q*_*j*_ decreases. As a solution, the *Q*-level could be lowered, or as an alternative, an iterative approach (e.g. [2]) could be performed, eliminating the GD with lowest *Q*_*j*_ in a first step and then perform QC again with the remaining GD.

In [6], a threshold of 0.8 was suggested. Lower thresholds could lead to unidentified corrupted GD and by a higher threshold, the Q-level of the whole data set will be lowered in data sets with more corrupted GD since Q-values of all volumes are influenced by each other.

That way, an artefact correction was performed by detecting GD with at least one slice showing intensity changes, i.e. artefacts caused by spontaneous subject movement or other sources of distortion.

### Impact of QC-based elimination of corrupted GD

**Figure 2:**
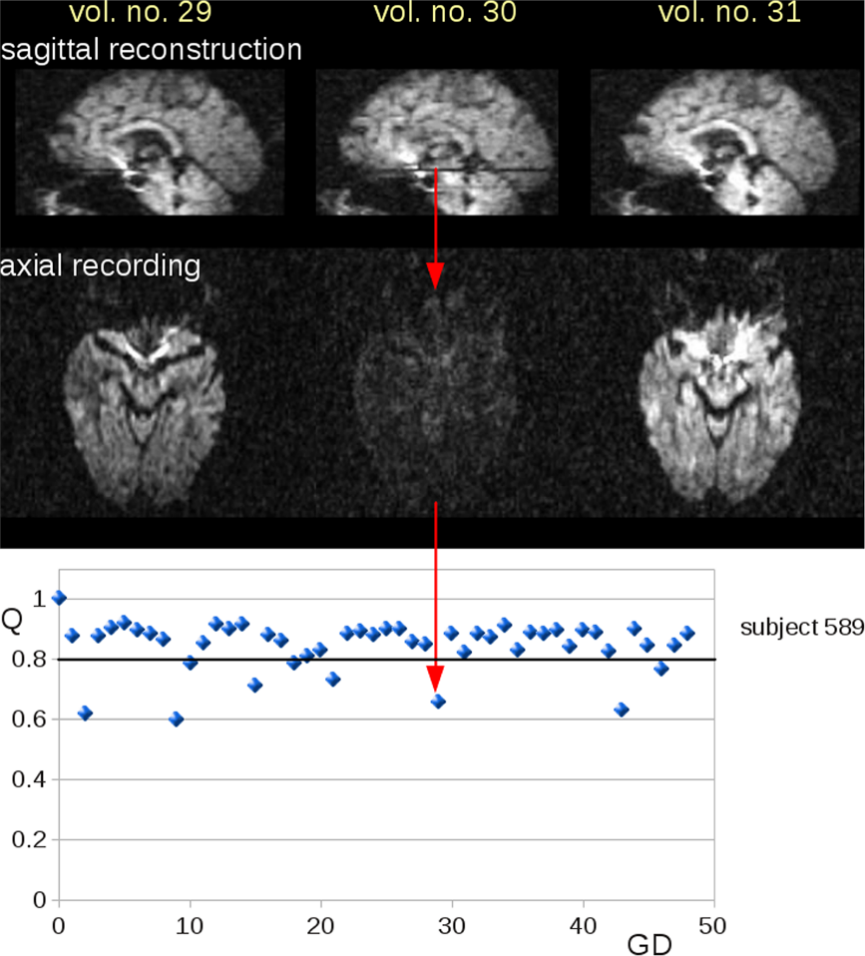
**Corrupted slices in DTI volumes.** DTI data (volumes number 29 to 31) of an HD subject (subject 589). **Upper panel:** reconstructed central sagittal slice where axial slices affected by noise are visible (volume number 30). **Lower panel:** With a QC-threshold of Q = 0.8, nine gradient directions (GD) were detected as corrupted by noise.

The impact of QC-based elimination of corrupted GD was investigated in two ways:

1. The possible impact of corrupted GD on FA-maps was analysed by ROI analysis in FA maps prior to MNI normalization for a single visit with and without QC based elimination of GD. ROIs at the identical anatomical position of other visits could act as a reference. This analysis was performed by the following procedure: (i) DTI data of a single HD subject were selected in which two visits showed no corrupted GD and one visit showed a series of corrupted GD. (ii) A difference map was calculated from FA maps of the visit with corrupted GD by simple subtraction of the FA maps with and without GD elimination. (iii) In a data-driven approach, a ROI was placed in a brain region where differences in FA were detected by the difference map. (iv) An experienced operator identified the anatomical ROI position in the FA maps of the other visits without the knowledge of FA values. (v) Mean FA values of all visits in the respective ROIs were compared in the contrast with vs. without QC-based GD elimination.
2. Using cross-sectional whole brain comparison the impact of corrupted GDs on FA maps was assessed in HD participants and controls. Corrupted GDs may impact FA maps in two ways. (i) FA values differ (Figure  1A and Figure  2) and (ii) MNI Registration of the subject’s FA image to the study-specific group template (which are based on study specific FA templates calculated from single subject FA maps) also could show differences, i.e. the MNI normalization process of all DTI data sets might also be influenced by differences in the FA maps of individual participants (Figure  1B).

Furthermore, between-group differences in cross-sectional data were investigated. The result patterns were compared to cross-sectional comparisons of DTI data sets from two further visits. Thus, each time point of the longitudinal data has been used as a single cross-sectional test point. This way, longitudinal data per-subject was used as a scan-rescan reproducibility test with the inherent assumption that the effect of progression of the disease over the time scale of the study on the diffusion images is negligible in first order, and hence the group-wise differences should only slightly proceed for the three time points.

## Results

### Impact of corrupted GD on individual FA maps

Hypointense slices indicating corruption in single volumes of an individual DTI data set (Figure 2, upper panel) were identified by reduced *Q*-values (*Q* < 0.8) within the respective volume (Figure 2, lower panel).

**Figure 3:**
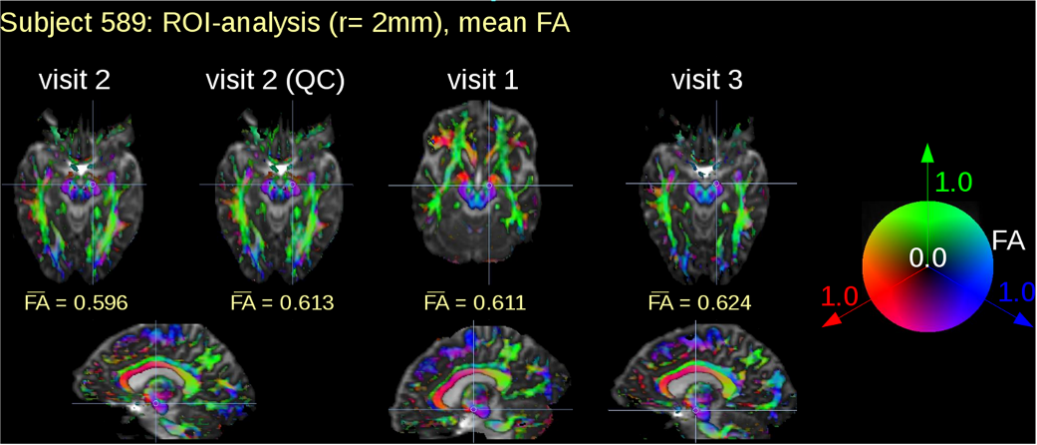
**Region of interest analysis of FA maps from three longitudinal DTI scans.** Region of interest (ROI) analysis of visit 1 and visit 3 were without gradient direction (GD) elimination by quality control (QC) since no corruption was evident. During visit 2, 9 GD (compare Figure 1) had to be eliminated. ROI based mean FA values changed with and without QC, respectively. Although ROI localization was identical for the three visits the respective slices look apparently different due to different slice orientation during acquisition.

In more than 50% of the HD patients’ DTI data, a constellation could be found where GD elimination led to an adjustment of ROI-based mean FA values. Figure 3 illustrates an example for a ROI analysis (HD subject 589). Here, visits 1 and 3 had no GD eliminations while nine corrupted GD were found in visit 2. A ROI was placed in the position where the difference matrix of visit 2 (with vs. without GD elimination) showed its local maximum in FA differences. Mean FA values in the ROI were compared to mean FA values of visits 1 and 3 at the identical ROI positions. Thus, without QC, FA values in the selected ROI in FA maps from visit 2 data were lower compared with FA values from visits 1 or 3. After elimination of the nine corrupted volumes, FA values of visit 2 data in the selected anatomical ROI were identical to FA values of visits 1 and 3.

### Impact of corrupted GD on cross-sectional comparison of FA maps

In the next step, the impact of QC-based GD elimination on the cross-sectional comparisons between FA maps from HD participants against controls was investigated: results clusters, i.e. patterns of significant FA reductions and increases, from cross-sectional differences between HD subjects and controls were compared with or without QC-based GD elimination for the three visits.

**Figure 4:**
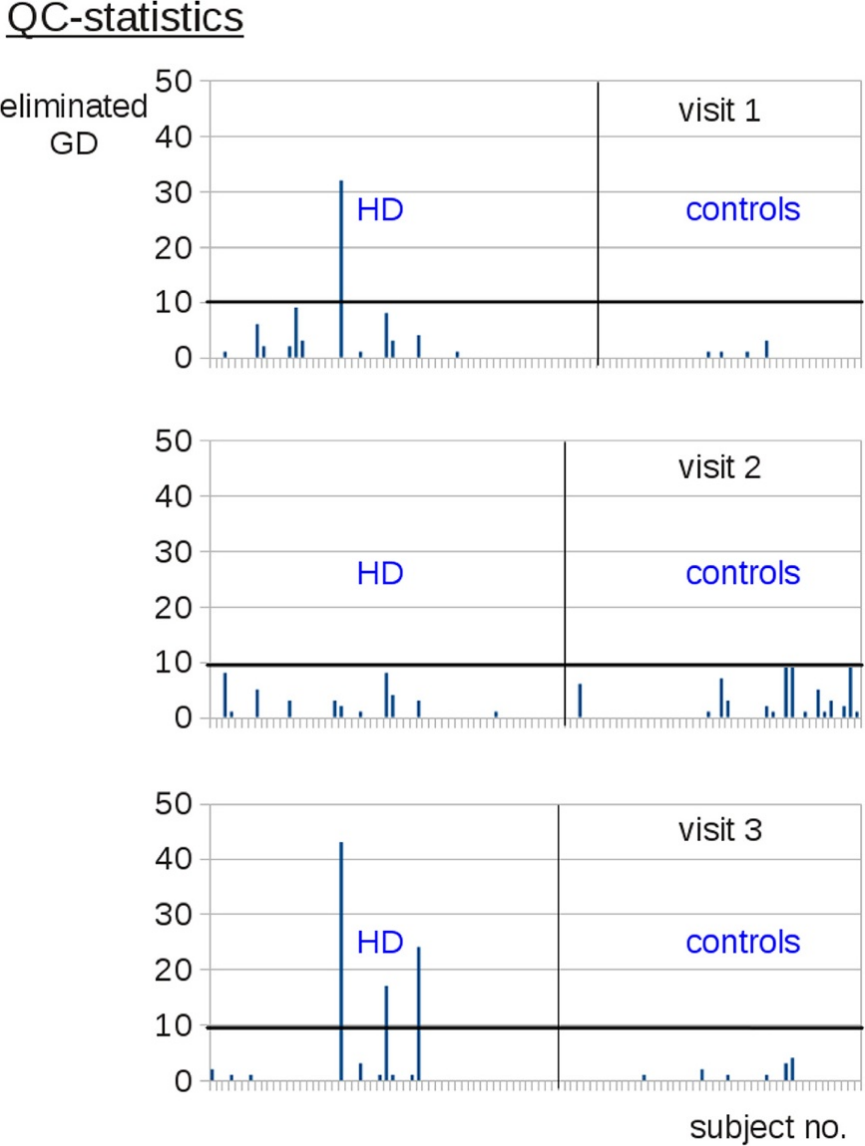
**Quality control statistics.** Gradient direction (GD) elimination statistics per each visit displays the number of gradient directions excluded in the QC process separately for each subject and for each of the diagnostic groups.

The application of the QC algorithm resulted in specific QC characteristics for each DTI data set with identified volumes to be eliminated for FA calculation. The statistics for the number of eliminated volumes for all DTI data sets are summarized in Figure 4 as GD elimination statistics. The numbers of GDs excluded during the QC process for each participant are displayed separately for each visit. The removed GD were tested on systematic distribution concerning (i) the frequency of a specific GD and (ii) the frequency of spatial orientation. A random frequency and spatial distribution of removed GD was found without clustering in a particular orientation, that way vibration-induced artifacts (e.g. [2]) could be excluded.

Four data sets showed more than 10 corrupted GD. An intrinsic property of the QC algorithm is that it shows a general reduction of the *Q* value for a greater number of corrupted GD. For these four scans the *Q* level was lowered to 0.7. Consequently, in each of the four scans more than 20 GD could be used for tensor calculations. Since this number is still sufficient due to the results of [6], the affected scans were not entirely eliminated from the study. All remaining scans showed less than 10 QC eliminations, i.e. less than 20% of GDs were eliminated in DTI data sets due to QC so that no (substantial) single data set FA changes could be expected according to [6].

**Figure 5:**
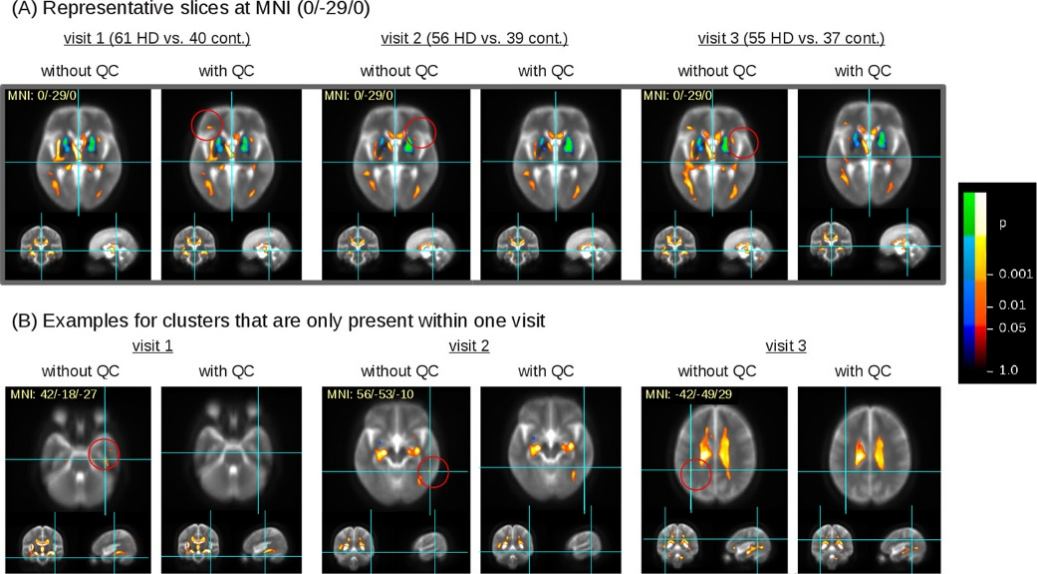
**Results of cross-sectional group comparison.** Clusters of significant FA differences between HD subjects and controls for the three visits. Hot colors indicate FA reductions in the comparison controls vs. HD subjects, cold colors indicate FA increases in this comparison. **(A)** Representative slices of identical MNI coordinates (0/-29/0) for visits 1 to 3 with and without quality control (QC), respectively. Red circles indicate additional clusters depending if QC based gradient direction elimination was performed, or not. **(B)** Examples of clusters that are only present in one visit, depending if QC based gradient direction elimination was performed or not. An example is shown for each visit 1 to 3; for clarity of presentation, the respective slices of the other visits for which no clusters could be detected are not displayed.

Cross-sectional result patterns differ if QC-based GD elimination was applied (see for example Figure 5, left panel – visit 1). The QC-based GD elimination influences the FA maps and thus, as a consequence, appearance of result clusters of smaller extent from cross-sectional group comparisons. For example, in visit 1 the group comparison without QC-based GD elimination showed a cluster with FA decrease in the hippocampal region (no. 21 – Table 1) which does not appear under QC-based GD elimination (type I error). On the other hand, a cluster in the frontal lobe (no. 14 – Table 1) appears for visit 1 with QC-based GD elimination which is not present without QC-based GD elimination (type II error). The hippocampal cluster (no. 21) was not confirmed by longitudinal data, i.e. cross-sectional group comparisons at visits 2 and 3, whereas the frontal lobe cluster (no. 14) appears also at visit 2 and visit 3 cross-sectional comparisons.

**Table 1:** **Cluster statistics for cross-sectional group comparison**

cl.			Visit 1	Visit 2	Visit 3	Visit 1 (QC)	Visit 2 (QC)	Visit 3 (QC)
1	basal ganglia	R	4,337	5,143	4,491	4,579	5,356	4,551
	MNI (x/y/z)		20/-6/-2	18/-7/-2	20/-7/-1	21/-6/-2	19/-8/-2	23/3/3
2	basal ganglia	L	3,997	2,574	2,553	4,287	2,873	2,571
	MNI (x/y/z)		−19/-8/-3	−18/-7/-4	−25/2/7	−19/-8/-3	−25/3/7	−24/3/6
3	thalamic region internal capsule	R/L	40,800	1,617	84,048	7,961	1,665	5,560
	MNI (x/y/z)		34/-17/-13	34/-18/-11	35/-19/-12	35/-18/-12	34/-18/-11	35/-18/-12
4	thalamic region	R	within cl. 3	within cl. 11	within cl. 3	32,070	1,289	within cl. 11
	MNI (x/y/z)					−3/-14/4	−19/-10/9	
5	thalamic region	R	within cl. 3	within cl. 11	within cl. 3	1,859	within cl. 11	within cl. 11
	MNI (x/y/z)					25/4/-13		
6	thalamic region	R	1,072	within cl. 11	within cl. 3	738	within cl. 11	within cl. 11
	MNI (x/y/z)		16/-40/4			16/-41/4		
7	thalamic region	L	13,094	4,770	within cl. 3	11,640	2,743	4,622
	MNI (x/y/z)		−21/-21/-15	−20/-22/-13		−21/-21/-13	−20/-22/-13	−23/-21/-11
8	internal capsule	L	within cl. 7	432	within cl. 3	within cl. 7	335	within cl. 11
	MNI (x/y/z)			18/-9/12			18/-9/12	
9	external capsule	L	within cl. 7	within cl. 11	within cl. 3	within cl. 7	1,406	within cl. 11
	MNI (x/y/z)						−35/-15/7	
10	external capsule	R	255	within cl. 11	within cl. 3	272	within cl. 11	within cl. 11
	MNI (x/y/z)		36/-13/8			34/7/2		
11	CC	R/L	1,400	41,508	within cl. 3	3426	35,008	37705
	MNI (x/y/z)		−9/18/12	−11/-25/25		−7/17/13	−11/-25/25	−27/-56/19
12	occipital lobe	R	2,210	2,026	3,166	2,160	2,015	1,845
	MNI (x/y/z)		35/-89/-8	38/-74/-5	38/-71/-7	34/-88/-8	38/-75/-4	37/-73/-6
13	occipital lobe	L	1,685	1,027	within cl. 3	2,010	950	891
	MNI (x/y/z)		−40/-68/2	−39/-69/2		−41/-68/1	−39/-69/2	−40/-67/1
14	frontal lobe	L	no cluster	294	497	373	280	453
	MNI (x/y/z)			−34/27/6	−41/29/-1	−40/29/1	−34/27/6	−41/29/-1
15	limbic lobe	L	no cluster	no cluster	within cl. 11	296	422	within cl. 11
	MNI (x/y/z)					−16/0/37	−15/3/35	
16	parietal lobe	L	682	no cluster	within cl. 11	within cl. 3	within cl. 11	within cl. 11
	MNI (x/y/z)		−18/-49/34					
17	occipital lobe	L	643	319	within cl. 3	283	307	within cl. 13
	MNI (x/y/z)		−22/-81/22	−22/-80/24		−23/-82/22	−22/-80/23	
18	temporal lobe	L	205	no cluster	within cl. 3	172	1279	203
	MNI (x/y/z)		−46/-52/3			−47/-50/3	−48/-53/5	−46/-51/7
19	parietal lobe	R	230	no cluster	311	256	no cluster	317
	MNI (x/y/z)		19/-64/49		19/-61/47	19/-62/50		18/-63/49
20	internal capsule	R/L	195	no cluster	no cluster	no cluster	no cluster	no cluster
	MNI (x/y/z)		−10/6/-6					
21	hippocampus	R	192	no cluster	no cluster	no cluster	no cluster	no cluster
	MNI (x/y/z)	L	−26/-20/-26					

Clusters (cl.) of the FA map group comparison between HD subjects and controls (cl. size/voxels and MNI coordinates of the area with highest significance). All clusters listed in this table show a p-value of p < 0.001. Basically, several cluster groups that are interconnected in different analyses were found. Clusters 1,2 (FA increase): clusters located in the basal ganglia. Clusters 3-17 (FA reduction): clusters covering the thalamic area, external and internal capsule, and corpus callosum (CC); additional clusters in the occipital, frontal, parietal lobe, and limbic regions. Clusters 18-21 (FA reduction): small clusters in the temporal and parietal lobe, the internal capsule, and the hippocampus.

Figure 5 and Table 1 summarize results of cross-sectional group comparisons of HD participants and controls for the three visits with or without QC-based GD elimination. The overall patterns of FA reductions or increases were consistent for all visits, i.e. regional FA increases in the basal ganglia and FA reductions in the internal and external capsule, thalamic regions and corpus callosum (CC). This result was achieved although the number of contributors differed during each visit. For the three visits, cross-sectional FA comparisons showed result patterns sharing common clusters. This effect also appeared irrespective of the analysis being performed with or without QC-based GD elimination; as a consequence cluster locations and sizes in Table 1 could not be directly compared. FA reduction clusters covered the internal and external capsule, the thalamic region, and the CC. In addition, clusters were found in the occipital, frontal, parietal lobe, and limbic areas. Clusters with FA increase involved the basal ganglia. Depending on the performance of QC-based GD elimination, additional small clusters appeared in the temporal and parietal lobe, the internal capsule, and in the hippocampus.

Cluster 18 is an example of a cluster that does not show up in visit 2 (cross-sectional comparison) without QC (type II error). Clusters 20 and 21 are examples of clusters that appear only in visit 1 without QC (type I error).

## Discussion

### Effect of corruptions in a DTI data set

Movement associated image corruptions are a matter of concern in neuroimaging research. These artefacts may reduce the potential of DTI as a biomarker in HD. Algorithms are therefore needed that compensate for corrupted volumes in a DTI scan.

In this study, we used an algorithm for automatic and objective detection of volumes that contain corrupted slices. Identified volumes with corrupted GD were then excluded from further calculations of DTI metrics. The question to what extent GD could be removed from a DTI data set without relevant changes to FA maps had previously been addressed [6]. The present study is a continuation of QC-based studies of our group as well as of other groups [2, 4, 5, 7]. It could be demonstrated in the present study that corrupted volumes in DTI acquisitions have impact on the results of cross-sectional group comparisons of FA maps.

The effects of corrupted volumes on cross-sectional result patterns were two-fold: first, corrupted volumes influenced FA values alone, and secondly, as a consequence, they could have impact on registration of the subject’s FA image to the study-specific group template (which is based on study specific FA templates calculated from single subject FA maps). This means that the MNI normalization process of all DTI data sets was also influenced by differences in the FA maps of individual participants.

In this study, no entire scans had to be eliminated although the first QC showed some scans with a greater number of corrupted GD. For these cases, a Q threshold reduction could detect the most corrupted volumes and the number of remaining GD was considered still sufficient for accurate tensor detection. In cases where Q threshold reduction detects so many GD that only few non-corrupted GD would survive correction, we suggest that such a data set should be entirely eliminated from the study.

### Application of QC-based GD elimination on cross-sectional result patterns

The results of our analysis demonstrate that the voxelwise pattern of cross-sectional FA group differences between HD subjects and controls showed high consistency with previous reports of other research groups (e.g. [10, 11]). In addition, the repeated cross-sectional group comparisons showed rather constant result patterns. Differences between results with and without QC-based GD elimination in terms of cluster size and location originate from interconnections between clusters.

Importantly, the presence or absence of clusters in FA maps of visit 2 relative to visits 1 and 3 most likely reflect type I or type II errors, respectively, since their localisation does not match with brain regions typically affected in HD whose FA changes could be explained by the course of the disease per se. Since some of the cross-sectional FA changes of visits 1 and 3 are consistent, differences in FA changes for visit 2 cannot be reasonably attributed to disease progression, due to the temporal relationships between the data. In a single cross-sectional test point, the inconsistency of clusters between the group-wise results with and without corrupt GD removal is not technically adequate to label the differential presence or absence of clusters as type I or type II errors: however, the consistency of these results across three separate cross-sectional tests provides additional evidence for classification.

### Limitations of the study

This study is based on a general approach how the incorporation of corrupted GD could affect tensor calculations and group differences at whole brain level and at the group level. However, if the gradient directions removed are isotropically distributed both within and between subjects, the influence will be akin to SNR reduction. If gradients in a particular direction are more likely to lead to artefact and hence volume removal (as it is the case in vibration-induced artefact in diffusion imaging), this could cause a bias in tensor estimation, and therefore non-stationarity in statistical power depending on the underlying fibre orientations. It is open to discussion whether the whole volume should be eliminated when only a few slices show corruptions. If only single slices were omitted for FA calculation, the remaining voxels (with no slice in any volume being corrupted) would show less bias. On the other hand, FA maps would then be calculated where the voxel FA values in one FA map would originate from a different number of GDs. In order to keep the number of GD constant from which FA values of an individual FA map are calculated, we suggest that each FA map should be calculated with the number of GD contributing to the diffusion tensors in the voxels should be kept constant for individual FA maps if repeatedly measured across time. Nevertheless, FA maps of different subjects could still originate from different sets of GD.

A different situation emerges regarding intraindividual longitudinal data comparisons. Here, case one would require the number of GDs to be identical across repeated measurements for single subjects to reliably estimate longitudinal changes in FA maps. For the aim of this study, longitudinal data were only used for validation of cross-sectional result patterns while we did not compare FA maps within HD subjects, or controls, longitudinally. Therefore, further research is needed how to apply QC on GD for longitudinal FA analysis.

With respect to the validation of cross-sectional result patterns by longitudinal data itself, it might be considered that the consistency of these clusters between the three time points (or lack thereof) does not in all cases provide evidence for the labelling of these inconsistent clusters as errors. In fact, if a cluster is present without GD rejection and disappears with GD rejection, this suggests but is not full evidence to label this cluster as a type I error, as the ‘actual’ difference in unknown; it is the absence of this cluster in the statistical tests at other time points that makes it probable that the cluster is in fact a statistical error.

The QC-based GD elimination described in this study is a global tool to eliminate corrupted GD. However, if slice dropout is caused by subject movement, the probability of the subject moving back to the precise location they were in before the movement is rather unlikely. Therefore, even explicit inter-volume motion correction may not be adequate to fully recover these volumes, as non-linear volume components will be present in the movement regression. This depends on factors like slice timing and order. Therefore, the ultimate justification for rejection of GD is grounded in the mechanism by which diffusion images are acquired.

## Conclusion

In summary, QC of DTI data clearly impacted upon the results pattern of FA reductions and increases at the group level when comparing HD subjects and controls. We could show that some small clusters were observed in cross-sectional results without QC-based GD elimination, suggesting that corrupted GDs may increase the risk of type I errors and should consequently be eliminated during a QC process. Furthermore, QC-based GD elimination was associated with emergence of otherwise undetected clusters of group differences indicating that QC-based GD elimination may also protect against type II errors at the level of group comparisons.

## Abbreviations

FA: Fractional anisotropy
FDR: False discovery rate
GD: Gradient directions
HD: Huntington disease
MNI: Montreal Neurological Institute
ROI: Region of interest
SNR: Signal to noise ratio
TIFT: Tensor imaging and fiber tracking
TFC: Total functional capacity
UHDRS: Unified Huntington’s disease rating scale
QC: Quality control.
